# Latroeggtoxin-VI improves depression by regulating the composition and function of gut microbiota in a mouse model of depression

**DOI:** 10.1099/jmm.0.001977

**Published:** 2025-04-09

**Authors:** Haiyan Wang, Zhixiang Lei, Yiwen Zhai, Minglu Sun, Si Chen, Panfeng Yin, Zhigui Duan, Xianchun Wang

**Affiliations:** 1 State Key Laboratory of Developmental Biology of Freshwater Fish, Protein Chemistry Laboratory, College of Life Sciences, Hunan Normal University, Changsha 410081, Hunan, PR China

**Keywords:** depression, depression mouse model, depressive behaviour, gut microbiota, latroeggtoxin-VI

## Abstract

**Introduction.** Depression has become one of the mental diseases that seriously affect human health. Its mechanism is very complex, and many factors influence the condition. An imbalance of the gut microbiota is being considered as a factor that impacts the occurrence and progression of depression. Future therapies may therefore tap into this connection, treating depression through manipulation of the gut microbiome.

**Hypothesis/Gap Statement.** Latroeggtoxin-VI (LETX-VI), a proteinaceous neurotoxin from *Latrodectus tredecimguttatus* eggs, was previously demonstrated to inhibit excessive inflammation and improve depression behaviours, suggesting that it might be able to regulate the balance of gut microbiota. The aim of this study was to explore the effects of LPS and LETX-VI on depressive behaviours and gut microbiota and to analyse correlations between changes in the gut microbiota and depressive behaviours.

**Methodology.** A murine model of depression was established, and the effects of LPS and LETX-VI treatment on depressive behaviours and gut microbiota were investigated.

**Results.** In the murine model, depressive behaviour was induced by LPS; the ratio of *Firmicutes* to *Bacteroidetes* (F/B) and the number of pro-inflammatory bacteria in the gut microbiota increased (*P*<0.01), while butyric acid-producing bacteria with anti-inflammatory effect decreased (*P*<0.05). Furthermore, the metabolic function of the gut microbiota was disrupted, and the level of virulence factors among gut microbiota was up-regulated (*P*<0.05). Association analysis showed that the changes in the composition and function of gut microbiota were closely related to the depression phenotype of mice, suggesting that the abnormal function of gut microbiota is linked to depression. However, when LETX-VI was applied before LPS injection, the LPS-induced changes in the gut microbiota were alleviated, and the depressive behaviour greatly improved.

**Conclusion.** LETX-VI can prevent depressive behaviour by regulating the composition and/or function of the gut microbiota.

## Introduction

The prevalence of depression has soared, and depression has become one of the mental diseases that seriously affect human health in today’s society. Depression is a highly heterogeneous disease that is often comorbid with other diseases; different patients respond differently to the same treatment plan, resulting in misdiagnosis and low efficiency of treatment, making depression one of the problems that need to be solved urgently [1,4].

The aetiology of depression is very complex. In addition to many other factors that influence this condition, the imbalance of gut microbiota is also being considered as a factor that affects the occurrence and progression of depression. Studies have found that the gut pathogenic and inflammatory bacteria in patients with depression are significantly higher than those in healthy people, while the proportion of anti-inflammatory bacteria is significantly lower [2]. Such changes in the composition and function of gut microbiota further aggravate the psychiatric disorders, including depression [5,10]. When the ‘depression bacteria’ of depressed mice were implanted into healthy germ-free mice, they developed the same depressive-like behaviours. However, when the ‘healthy flora’ of healthy mice was implanted into healthy germ-free mice, the mice were still healthy and did not show depressive symptoms [11]. Gut microbiota can change the behaviours of mice through inflammatory pathways [12]. Long-term stress can lead to the activation of the immune system and a significant increase in inflammatory factors and thus depression. Meanwhile, there are studies indicating that acute stress can stimulate mast cells and increase the permeability of the gastrointestinal barrier and even the blood-brain barrier, which leads to the leakage of various substances metabolized by gut micro-organisms, such as toxins and LPS. These metabolites enter the body’s circulatory system and even the central nervous system, resulting in the aggravation of inflammatory response and depression [13]. Gut microbiota and the host have mutual regulatory effects. The gut microbiota can intervene in the host brain and induce depression by regulating the immune system, and the brain can also influence the composition and function of the host gut microbiota through the immune system [14,17]. The close relationship between gut microbiota and depression suggests that gut microbiota is an important target for the prevention and treatment of depression [10]. A series of pharmacotherapies are used to treat depression, and their mechanisms of action include regulating gut microbiota. For instance, fluoxetine is widely used for the treatment of depression, and oral administration of fluoxetine alleviated LPS-induced depressive behaviours and gut microbiota dysbiosis through multiple mechanisms, such as increasing the proportion of *Lactobacillus* in the gut microbiota [18]. When Sun *et al*. [19] investigated the potential connection between gut microbiota and anti-inflammatory action of schisandrin (SCH) on a depressive mouse model induced by LPS, it was found that the administration of SCH prior to LPS reduced the levels of pro-inflammatory factors, improved the depressive-like behaviours and attenuated the gut microbiota dysbiosis. Crocin-I, a major active component of crocin, was also found to exert antidepressant activity by mitigating gut microbiota dysbiosis [20]. Although there are a series of agents which indicate that depressive behaviours can be prevented or alleviated through modulation of gut microbiota, the underlying mechanisms are still not fully understood, and more effective drug candidates need to be explored.

Compared with small molecules, protein and peptide drugs have several advantages, such as higher bioactivity, specificity and safety [21,22]. Latroeggtoxin-VI (LETX-VI) is a proteinaceous neurotoxin found in the eggs of spider *Latrodectus tredecimguttatus*. Our previous study discovered that LETX-VI could protect nerve cells and prevent depression by inhibiting the activation of NF-кB signalling pathway and excessive inflammation [23], which aroused our interest in exploring whether the inhibitory action of LETX-VI on depression involves its influence on gut microbiota. In this study, we use a murine model of depression induced by intraperitoneal injection of LPS to study the effects of LPS and LETX-VI on depressive behaviours and gut microbiota and to identify correlations between changes in the gut microbiota and depressive behaviours.

## Methods

### Preparation of recombinant LETX-VI

For preparing recombinant LETX-VI with reference to the method previously described [24], total RNA was extracted from *L. tredecimguttatus* eggs, and cDNA was obtained by reverse transcription. The LETX-VI gene was amplified by nested PCR, cloned into the pET32a expression vector and transformed into *Escherichia coli* BL21 (DE3) for fusion protein expression. Ni-NTA beads were used for affinity purification of LETX-VI fusion protein. The purified LETX-VI fusion protein was enzymolysed with enterokinase, and the resultant LETX-VI was purified by reverse phase (RP)-HPLC and identified with MS. The obtained LETX-VI sample was freeze dried and stored at −80 ^°^C for later use.

### Development of depression mouse model and detection of LETX-VI affecting depressive behaviours

The 8-week-old male C57BL/6 J mice were used in this experiment. Each mouse was housed in a single cage in the specific pathogen free animal house and adaptively fed for 7 days before the experiment. The feeding environment was as follows: temperature 24±1 °C, relative humidity 40–60% and light cycle L 12 h:D 12 h.

The mouse model of depression induced by LPS was established according to the previous methods [25,27]. The male mice were randomly divided into three groups, control group, LPS group and LETX-VI-LPS group, each group consisting of ten mice. The mice in the control group were intraperitoneally injected with sterile saline; the mice in the LPS group were intraperitoneally injected with LPS (0.83 mg kg^−1^ body weight); the mice in the LETX-VI-LPS group were intraperitoneally injected with LETX-VI (3 mg kg^−1^ body weight) twice at an interval of 12 h, and 1 h later, LPS (0.83 mg kg^−1^) was injected. Twenty-four hours after LPS injection, behavioural tests, body weight detection and gut microbial assay were performed. The performed behavioural tests included sucrose preference test (SPT), tail suspension test (TST) and forced swim test (FST). These tests were commonly used in validating depression model, screening antidepressant drugs and evaluating other manipulations that are expected to render or prevent depressive-like states [28].

#### Sucrose preference test

The SPT was conducted by referring to the described methods [29,30]. The mice were raised individually in a cage and had free access to sucrose solution and food. Firstly, the mice underwent sugar water adaptation; namely, two bottles containing 1% sucrose solution were placed for the mice to freely drink; replace the sucrose solution in one of the bottles with pure water after 24 h, changing the position of the bottles every 12 h during this process to avoid location deviation. After a 2-day adaptation period, all mice were deprived of food, water and sucrose solution for 24 h. After drug administration, mice were allowed to freely access 1% sucrose solution and purified water for 24 h, during which the positions of 1% sucrose solution and purified water were changed to avoid position deviation at 12 h, and the sugar solution and purified water bottle were gently removed and weighed to determine the consumption amount of sucrose solution and purified water. Sucrose preference (%)=sucrose solution consumption (g)/[(sucrose solution consumption (g)+purified water consumption (g)]×100%.

#### Tail suspension test

The TST was carried out according to the method of Steru *et al.* [31]. Mice were acoustically and visually isolated, more than 15 cm away from the nearest object. The mouse tail was taped to a horizontal stick 1–2 cm away from the tail tip (~30 cm from the ground) so that the mouse was suspended head down. The mouse struggled to overcome the abnormal tail-hanging position and then appeared immobile or desperate. Each mouse was observed for 6 min, and its immobility time in the last 4 min was recorded to reflect its desperate state. Each mouse was removed immediately after the experiment and put back into its cage to roam freely.

#### Forced swim test

The FST was performed according to the methods of Wang *et al.* with minor modifications [32]. During the experiment, the mouse was held by grabbing its tail and gently putting the mouse into a glass tank filled with warm water; the temperature of the water was ~23–25 °C, and the water depth was ~10 cm. Each mouse was allowed to swim in the water for 6 min, and the stillness time within the last 4 min was recorded. Each mouse was tested separately, and the dirty water was replaced with freshwater after the completion of the test. The mouse was wiped with a dry paper towel, dried with a heat blower and returned to its cage after the test.

### Extraction of gut microbes from mice

After behavioural and body weight detection, the mice in the control group, LPS group and LETX-VI-LPS group were anesthetized with CO_2_ and killed. The extraction of gut microbes was aseptically operated on a super-clean workbench, and the fresh faecal samples from mice were collected by referring to the methods described in references [11,33]. The samples were snap frozen with liquid nitrogen and stored at −80 °C for later use.

### Metagenomic sequencing

An E.Z.N.A. DNA extraction kit (Omega Bio-Tek, USA) was used to extract mouse gut microbial DNA according to the instructions of the manufacturer. The integrity of the extracted DNA was detected with 1% agarose gel electrophoresis, and the concentration and purity were determined with a NanoDrop 2000 spectrophotometer (Thermo Fisher Scientific Inc., CA, USA). A Covaris M220 focused ultrasonicator (Covaris, Woburn, MA, USA) was used to fragment the extracted genomic DNA to ~400 bp in length. The resulting fragments were end repaired, A-tailed and ligated with adapters, followed by PCR amplification, quality inspection and paired-end sequencing that was completed on Illumina NovaSeq™ X Plus (Illumina Inc., San Diego, CA, USA) using NovaSeq X Series 25B Reagent Kit according to the manufacturer’s instructions. The NEXTflex™ Rapid-DNA Seq Kit (Bioo Scientific, Austin, TX, USA) was used to construct the paired-end libraries.

The raw reads were filtered by removing adaptor sequences, low-quality reads (length <50 bp or with average quality value <20) and host genome sequence contamination [34], and finally, clean data were obtained. Quality-controlled sequences were assembled using megahit v1.1.2. Then, Prodigal v2.6.3 was used to predict genes [35], and genes with length ≥100 bp were translated into aa sequences. The gene sequences were clustered with CD-HIT v4.6.1 [36] to construct non-redundant gene sets to analyse the similarities and differences between samples.

### Statistical analysis

The alpha diversity indices of microbial communities, including Chao, Shannon and Simpson, were calculated using Mothur software (v1.30.1) to analyse the abundance and diversity of the microbial communities. QIIME 1 software (v1.8.0) was used to analyse the beta diversity of microbial communities, with principal component analysis (PCA) being used to analyse the similarity and difference of community composition between the experimental groups using the vegan package [37]. Correlation network analysis was made using Python’s Networkx package. Statistical analyses were performed using GraphPad Prism 8 (San Diego, CA, USA), and data were presented as mean±sd. Differences between the two groups were analysed by independent sample t-test, and for comparisons of more than two groups, one-way ANOVA and post hoc Tukey test were used. For all statistical tests, *P*<0.05 indicates significance. All the experiments were performed at least in triplicate.

The study was performed in accordance with the recommendations of the Guide for the Care and Use of Laboratory Animals of the China National Institute of Health, and the Ethics Committees of Hunan Normal University ratified all the experiments with animals (2019, No. 23).

## Results

### Preparation and identification of recombinant LETX-VI

**Fig. 1.**
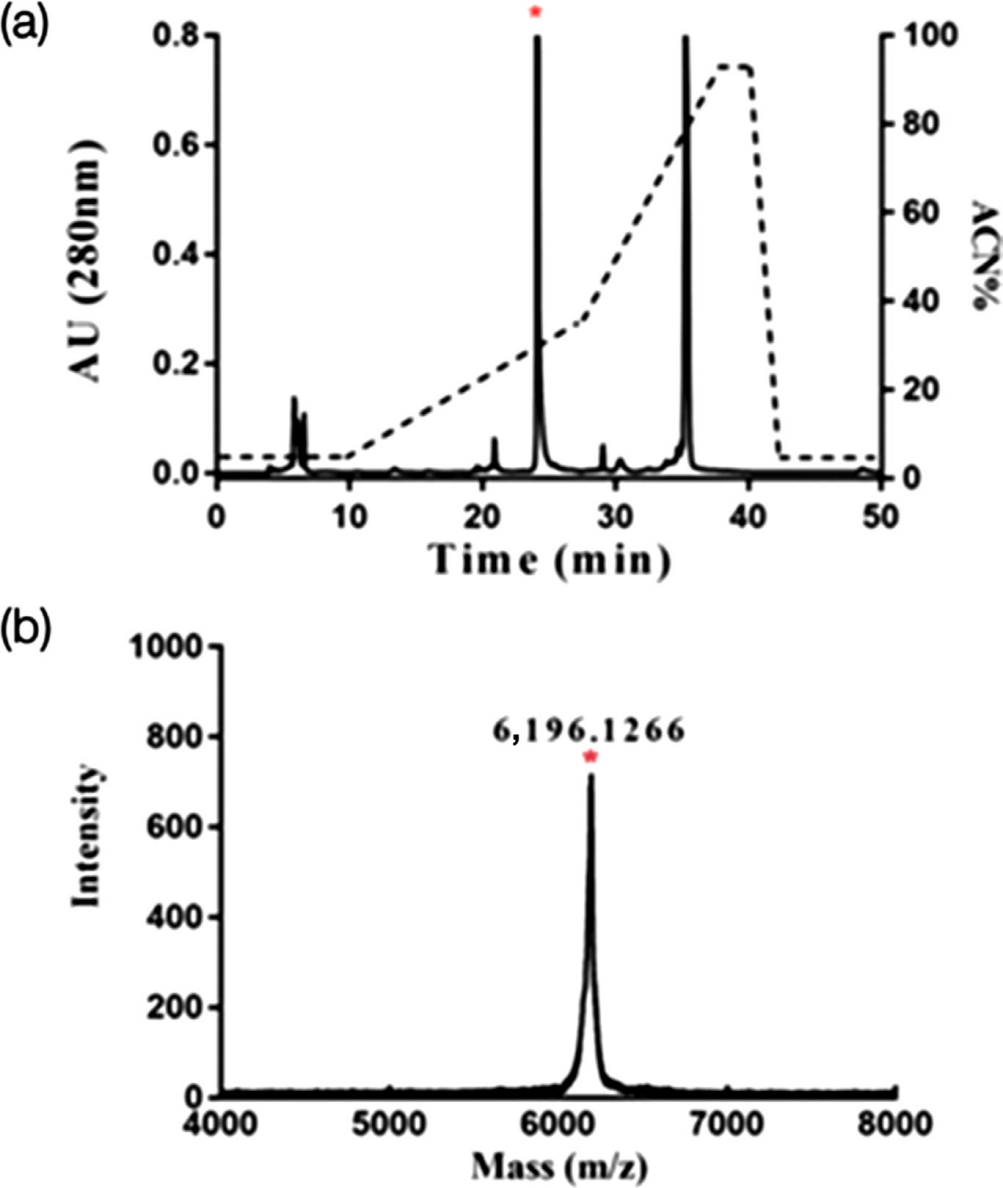
Preparation and identification of recombinant LETX-VI. (**a**) LETX-VI RP-HPLC separation results, the target peak marked by * in the figure. (**b**) MS identification of * labelled component in (a).

After heterologous expression in *E. coli*, the expressed LETX-VI fusion protein was affinity purified by nickel beads and treated with enterokinase, followed by separation by RP-HPLC (Fig. 1a). The peak of interest in Fig. 1(a) was marked with an asterisk, and the collected component was identified by MS (Fig. 1b). The monoisotopic molecular weight (M+H^+^) of LETX-VI was 6,196 Da, which is consistent with the mass of LETX-VI.

### LETX-VI improves depressive behaviours

**Fig. 2.**
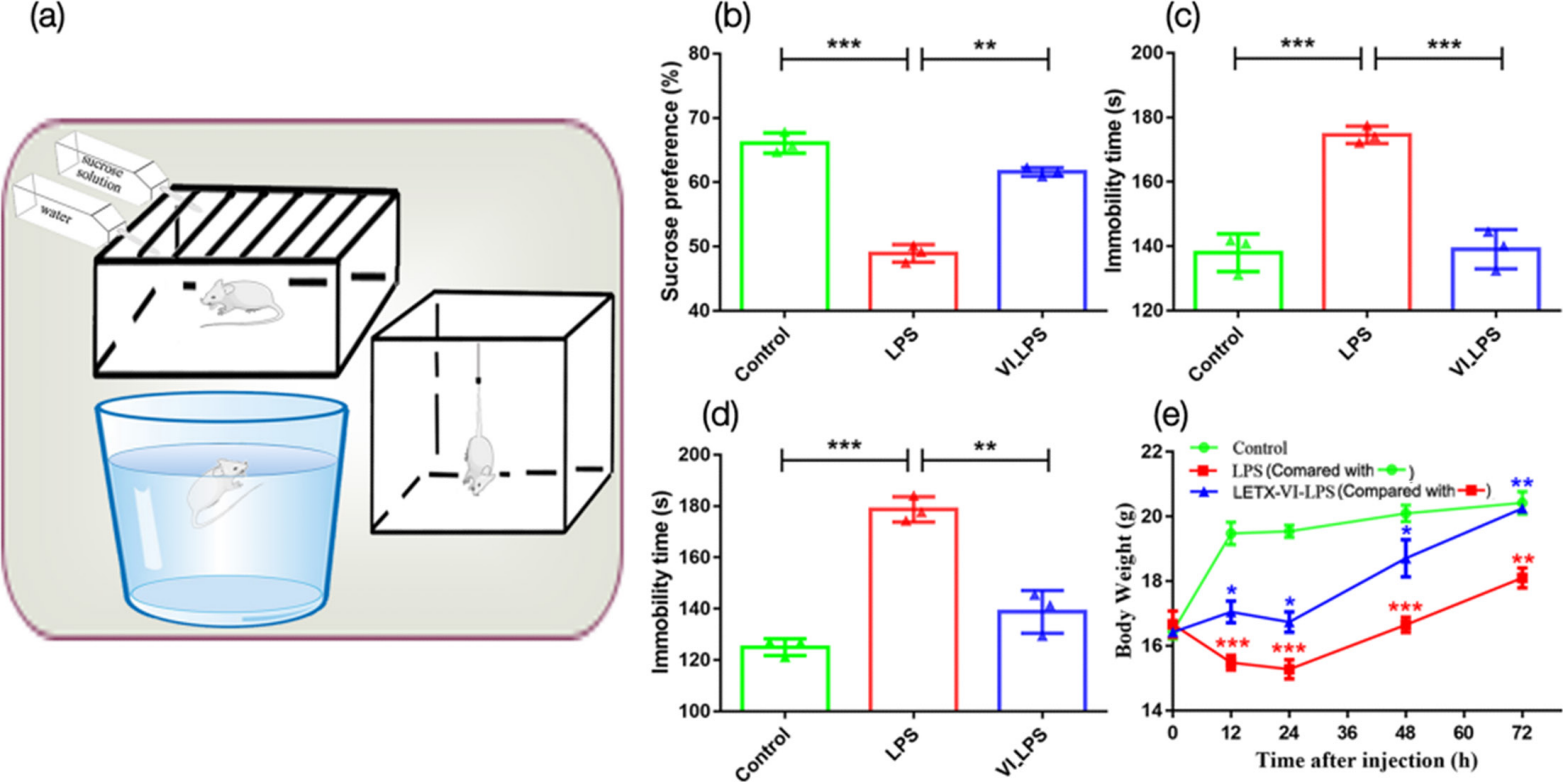
LETX-VI improves depressive behaviours and alleviates LPS-induced changes in body weight. (**a**) Behavioural test devices. (**b**) Effects of intraperitoneal injection of LPS and pretreatment with LETX-VI before LPS injection on the sucrose preference. (**c**) Effects of intraperitoneal injection of LPS and pretreatment with LETX-VI before LPS injection on the immobility times in TST. (**d**) Effects of intraperitoneal injection of LPS and pretreatment with LETX-VI before LPS injection on the immobility times in FST. (**e**) Effects of LPS and LETX-VI on the body weight of mice. LPS, LPS group; VI_LPS, LETX-VI-LPS group. *P<0.05, **P<0.01 and ***P<0.001.

After establishing the murine model for depression, we compared the depressive behaviours of the three groups of mice (Fig. 2a). Compared with the control, the LPS-induced mice had a significantly lower preference for sucrose solution (*P*<0.001, Fig. 2b) and a significantly increased immobility times in the FST (*P*<0.001, Fig. 2c) and TST (*P*<0.001, Fig. 2d), which is indicative of depressive behaviour [28]. However, mice in the LETX-VI-LPS group showed a significant improvement in the LPS-induced depressive behaviours (Fig. 2b–d), indicating a preventative effect of LETX-VI on the development of depressive behaviour in response to LPS. In addition, the body weight of the mice in the LPS group was significantly lower than that of the control group (*P*<0.001 or 0.01 at the test time points) and did not restore to the same level as that of the control 72 h after LPS injection. In contrast, the body weight of the mice in the LETX-VI-LPS group was significantly higher than that of the LPS group (*P*<0.05 or 0.01) and reached the same level as that of the control 72 h after drug administration (Fig. 2e). These findings are similar to our previous results [23] and confirm the effectiveness of LETX-VI pretreatment in attenuating the LPS-induced adverse influence on food intake and metabolism of substance and energy.

### Effects of LTEX-VI and LPS on *α*- and *β*-diversity of gut microbiota

After quality control of sequencing results, a total of 266,015,974 valid sequences (reads) were obtained, including 40,110,797,426 bases, and the average length of high-quality sequences was 150.78 bp. After removing redundancy, the number of genes was 6,413, containing 3,271,827 bp, and the average sequence length was 510.19 bp.

**Fig. 3.**
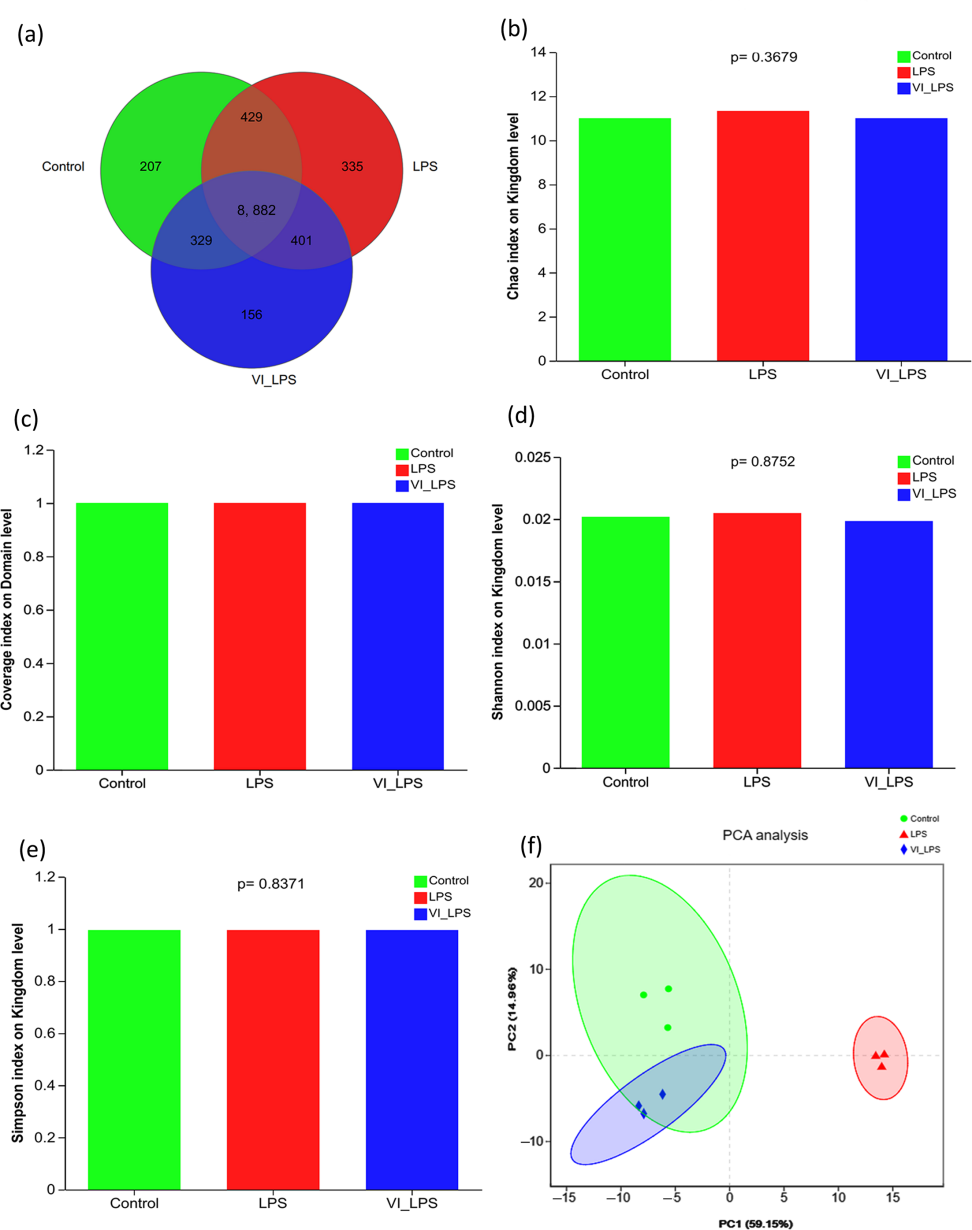
Diversity analysis of the gut microbiota in mice. (**a**) Venn diagram analysis of intestinal identified gut microbiota. (**b**) Chao index analysis of the gut microbiotas. (**c**) Coverage index analysis of gut microbiotas. (**d**) Shannon index analysis of gut microbiota. (**e**) Simpson index analysis of gut microbiota. (**f**) PCA of gut microbiota. LPS, LPS group; VI_LPS, LETX-VI-LPS group.

The Venn diagram for the number of identified micro-organism species (Fig. 3a) showed that the species distribution of the three groups of samples is mostly the same, but there are some differences among different groups. Among the identified 12,445 species, 8,882 species are common to the 3 groups and constitute the core micro-organism species of the gut microbiota; 207, 335 and 156 species are unique to the control group, LPS group and LETX-VI-LPS group, respectively, which means that LPS caused the most number of altered micro-organisms. Chao, coverage, Simpson and Shannon indices were used to measure the *α*-diversity and richness of gut microbiota in each group. As shown in Fig. 3(b–e), LETX-VI and LPS had no significant effect on these indices of gut microbiota in mice on the levels of kingdom and domain (*P*>0.05). *β*-Diversity analysis is mainly used in the comparison of differences in the overall structure of the microbial community of each sample, and the closer the distance between different groups, the more similar the microbial community. The PCA (Fig. 3f) showed that the LETX-VI-LPS and control groups were primarily concentrated on the left side, with partial overlap, whereas the LPS group was presented mainly on the right side, apart from both LETX-VI-LPS and control groups, suggesting that the gut microbiota from the mice in LETX-VI-LPS and control groups have similar overall structure, and that of the LPS group was significantly different. These results indicate that LPS treatment may cause changes in the gut microbiota of mice, and LETX-VI pretreatment can attenuate this effect.

### Effects of LTEX-VI and LPS on gut microbiota composition

**Fig. 4.**
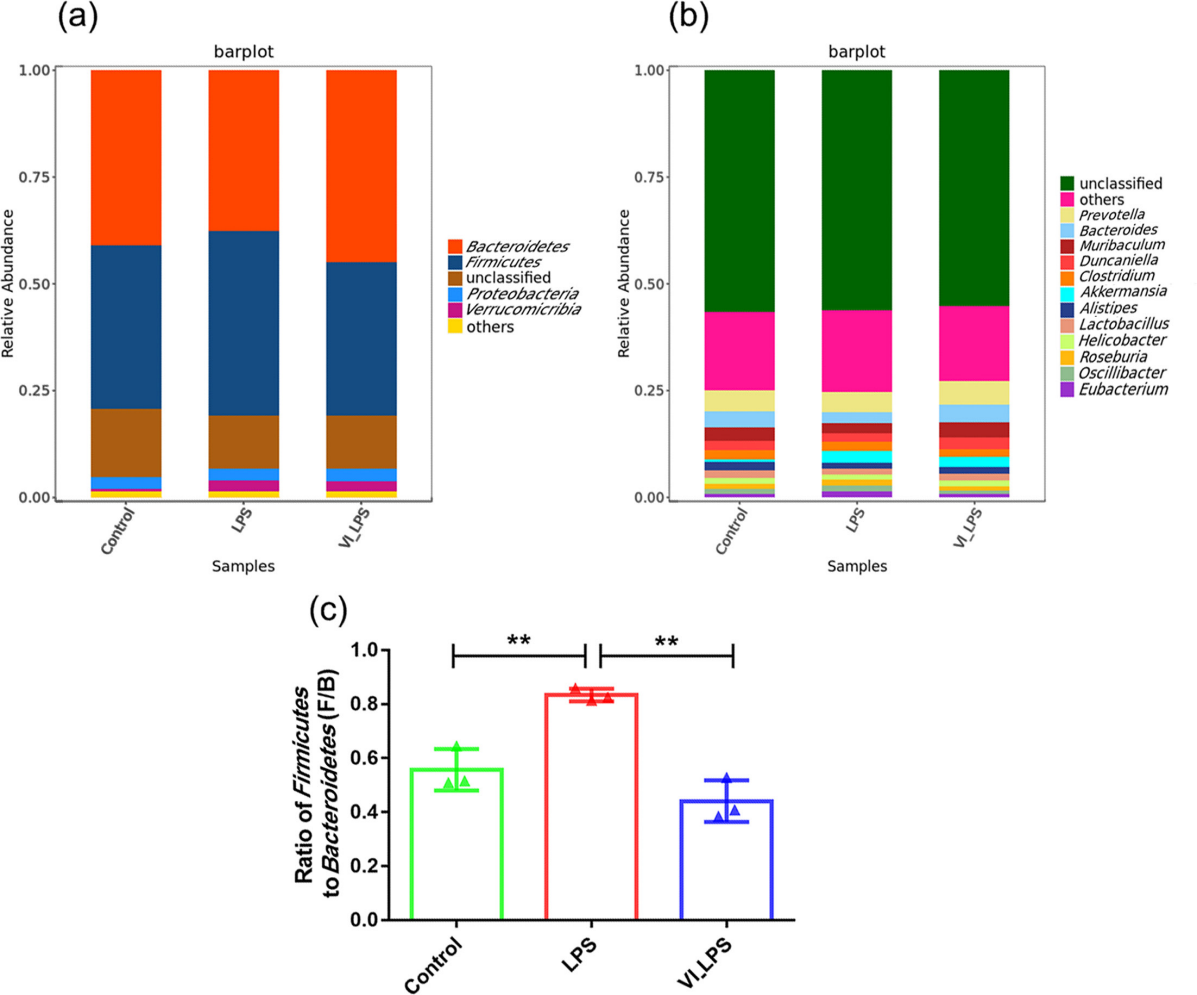
Composition analysis of mouse gut microbiota. (**a**) Column diagram of phylum-level composition of mouse gut microbiota. (**b**) Column diagram of the composition of mouse gut microbiota at the genus level. (**c**) *Firmicutes*/*Bacteroidete*s ratio of mouse gut microbiota. LPS, LPS group; VI_LPS, LETX-VI-LPS group. **P<0.01.

Through the comparison of different classification levels (phylum and genus levels) of gut microbiota in mice, the differences in microbial composition among the three groups of samples were analysed. As shown in Fig. 4(a), the analysis at the phylum level showed that the gut microbiota of the three groups of mice was mainly composed of *Bacteroidetes*, *Firmicutes*, *Proteobacteria* and *Verrucomicrobia*. *Bacteroidetes* and *Firmicutes* were the most abundant in each group of samples, followed by *Proteobacteria*. In addition, the relative abundance of *Bacteroidetes* was the highest in the LETX-VI-LPS group, while the relative abundance of *Firmicutes* was the highest in the LPS group. The analysis at the genus level (Fig. 4b) showed that *Prevotella* was the most abundant genus in all the samples, followed by *Bacteroides*, *Muribaculum*, *Duncaniella* and *Clostridium*. In addition, this study also found that, compared with the control, the ratio of *Firmicutes* to *Bacteroidetes* (F/B) in the LPS group increased significantly (*P*<0.01), whereas the F/B ratio significantly decreased in the LETX-VI-LPS group compared with the LPS group (*P*<0.01) (Fig. 4c). The above results demonstrate that the dominant phyla and genera of gut microbiota were similar in the three groups, but their relative abundance differed.

### Analysis of differential microbiotas in mice

**Fig. 5.**
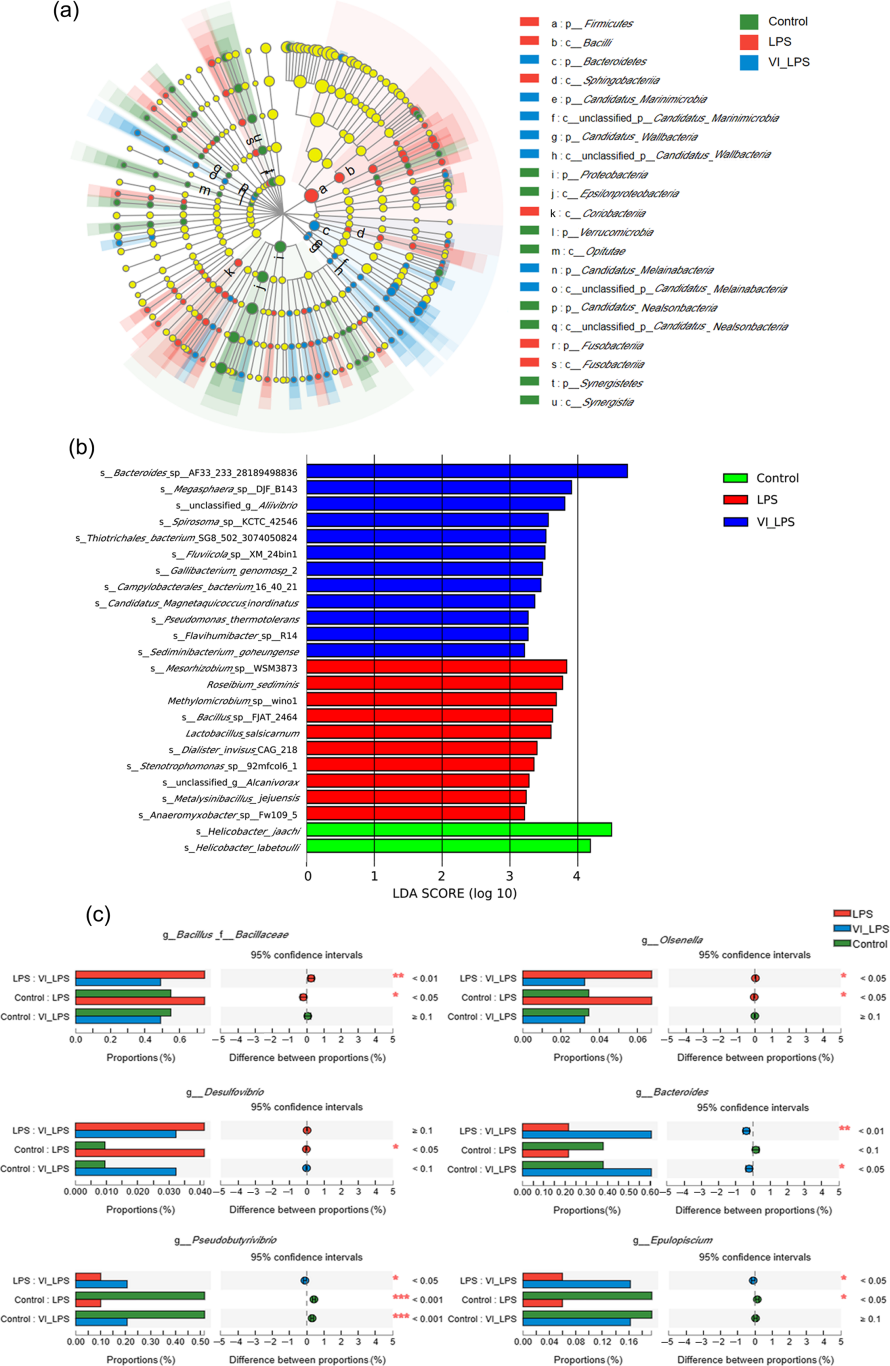
Analysis of differential microbiotas in the gut of differently treated mice. (**a**) LEfSe analysis of gut microbiota in mice. (**b**) LDA distribution histogram of differential gut microbiota in mice. (**c**) Multi-group comparison of differential bacterial genera in mouse gut microbiota. LPS, LPS group; VI_LPS, LETX-VI-LPS group. *P<0.05, **P<0.01 and ***P<0.001.

The linear discriminant analysis (LDA) effect size (LefSe) analysis was performed on the gut microbiota of the 3 groups of mice, and 149 micro-organism clades with significant differences in the levels from phylum to genus were confirmed (*P*<0.05) (Fig. 5a). Then, LDA was used to further analyse the differential bacterial species, and the resulting LDA histogram is shown in Fig. 5(b). The results showed that 12, 10 and 2 bacterial species were specifically enriched in the LETX-VI-LPS, LPS and control groups, respectively, including *Bacteroides*, *Megasphaera*, *Mesorhizobium*, *Roseibium* and *Helicobacter*.

There have been studies reporting that some bacterial species may transmit peripheral inflammation to the brain through the microbiota-gut-brain axis and play an important role in the occurrence and development of depression [4]. In order to understand the potential relationship between the different species of the three groups and the inflammation as well as depression and investigate whether LETX-VI could alleviate the LPS-caused disturbance of gut microbiota, we conducted multi-group comparison and significance analysis on the genus level. As shown in Fig. 5(c), compared with the control, the abundance of pro-inflammatory bacterial genera in the mice of the LPS group significantly increased (*P*<0.05), such as *Bacillus*, *Olsenella* and *Desulfovibrio*, whereas the abundance of these bacterial genera in the mice of the LETX-VI-LPS group was not significantly different. In contrast, the abundance of the bacterial genera that produce anti-inflammatory substances tended to decrease in the LPS-induced depression model mice compared with the control. These genera included *Bacteroides* (*P*<0.1), *Epulopiscium* (*P*<0.05) and *Pseudobutyrivibrio* (*P*<0.001). These observations suggest that LETX-VI may suppress gut inflammation and depressive behaviour through the microbiota-gut-brain axis by inhibiting or mitigating the adverse effects of LPS on some bacterial genera in the gut of the mice.

### Effects of LETX-VI and LPS on the metabolic functions of gut microbiota

**Fig. 6.**
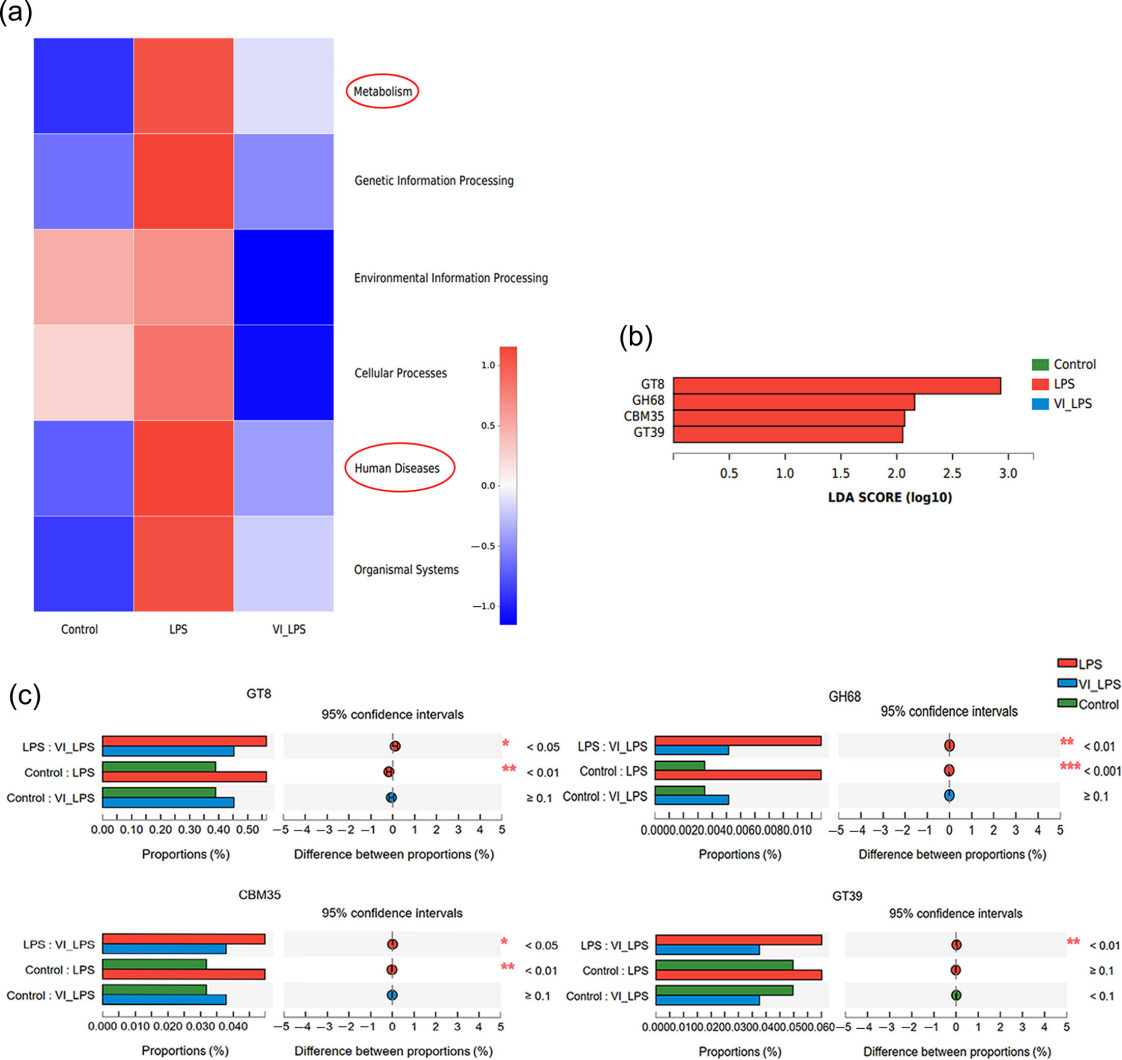
Effects of LETX-VI and LPS on gut microbiota function in mice. (**a**) Heat map analysis of KEGG function of gut microbiota in mice. (**b**) LDA score histogram of the content of the enzymes for carbohydrate metabolism in the gut microbiota of mice. (**c**) Multi-group comparison of the content of differential enzymes for carbohydrate metabolism in mouse gut microbiota. LPS, LPS group; VI_LPS, LETX-VI-LPS group. *P<0.05, **P<0.01 and ***P<0.001.

KEGG pathway enrichment analysis showed that all the six major categories of biological metabolic processes, including metabolism, genetic information processing, environmental information processing, cellular processes, human diseases and organismal systems, were affected by LPS to different degrees, and LETX-VI pretreatment efficiently attenuated the effects of LPS on metabolic processes (Fig. 6a). It is worth special noting that the metabolism and human disease pathways of LPS-induced depression model mice were significantly disrupted, and LETX-VI had a certain inhibitory effect on such LPS-induced disruptions, which suggested that the improvement of depression by LETX-VI treatment may be related to the reduction of disorders of metabolism and human disease pathways. We further analysed the metabolic pathways of gut microbiota in mice by ipath pathway map. The results showed that carbohydrate metabolism, aa metabolism, energy metabolism, lipid metabolism and some other metabolic pathways in mice were significantly affected. In particular, the analysis found that there were differences in the enzymes for carbohydrate metabolism among the three groups of gut microbiota samples. Compared with the control and LETX-VI-LPS groups, the LPS group was significantly enriched in glycosyl transferases 8 and 39 (GT8 and GT39), family 35 carbohydrate-binding module (CBM35) and glycoside hydrolases 68 (GH68) (Fig. 6b). Further analysis indicated that the content of these enzymes in the LPS group significantly increased compared with that of the control group, and LETX-VI pretreatment significantly inhibited the abnormal changes in the content of these enzymes induced by LPS, narrowing the gap between the LPS group and the control group (Fig. 6c). These results suggest that LPS may change the metabolic function of gut microbiota in mice, mainly involving carbohydrate metabolism, and LETX-VI displayed a certain ability to suppress these adverse changes.

### Effects of LETX-VI and LPS on virulence factors

**Fig. 7.**
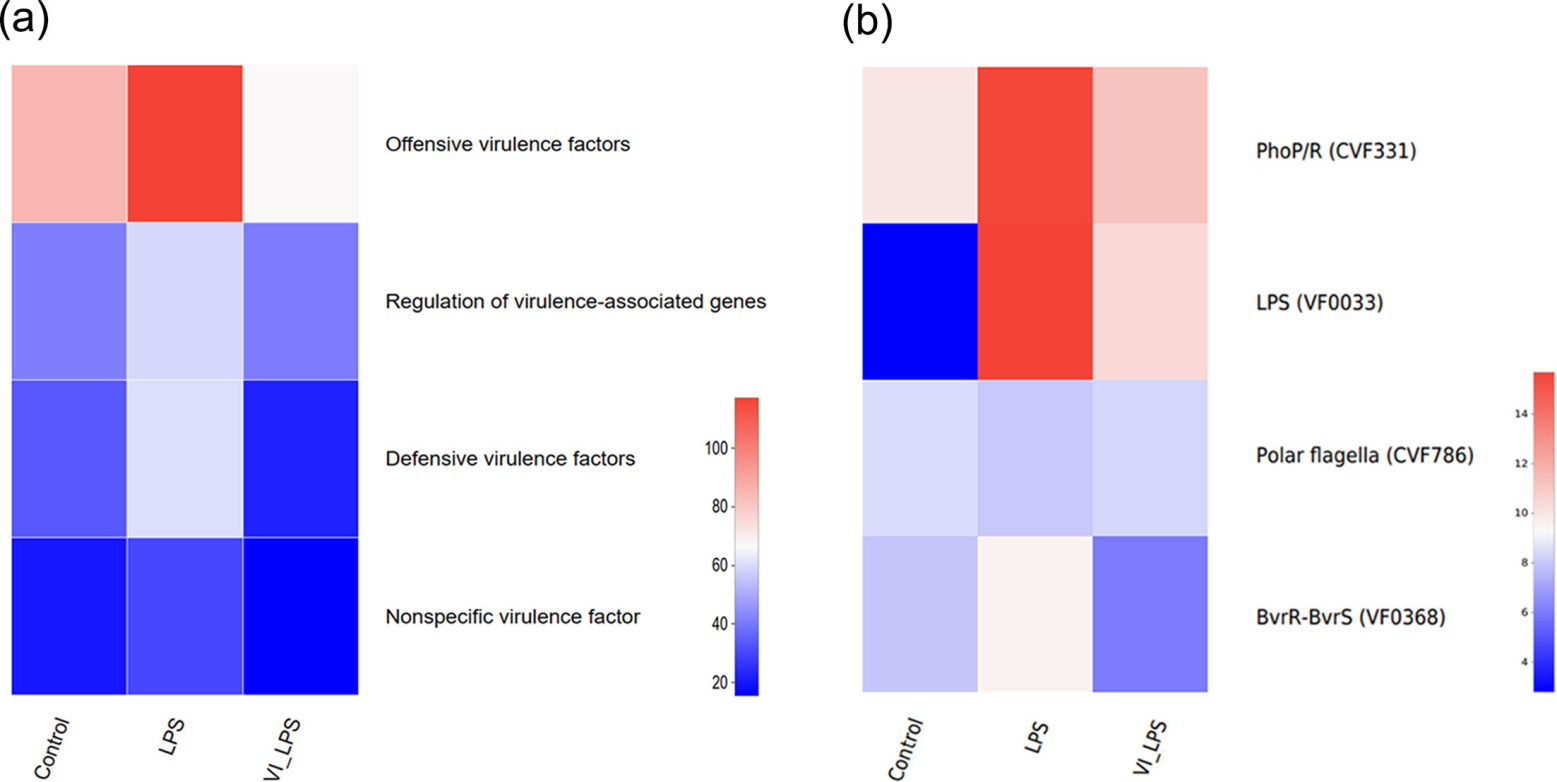
Effects of LETX-VI and LPS on virulence factors of gut microbiota in mice. (**a**) Heat map analysis of the changes in different classifications of virulence factors of gut microbiota in mice after LETX-VI and LPS treatment. (**b**) Heat map analysis of differential virulence factors in the gut microbiota of mice after LETX-VI and LPS treatment. LPS, LPS group; VI_LPS, LETX-VI-LPS group.

Some gut micro-organisms are able to adapt to the adverse environment in hosts by secreting a variety of virulence factors, which contribute to successful infection and cause disease [38]. Our analysis on the microbial virulence factors of the three groups of samples showed that, compared with the control, the gut microbiota of mice in the LPS group was enriched in bacteria that contain the genes for several groups of virulence factors, including offensive virulence factors, regulation of virulence-associated genes, defensive virulence factors and nonspecific virulence factors (*P*<0.05). However, LETX-VI pretreatment prevented or attenuated these enrichments (Fig. 7a), which suggest that LETX-VI may weaken the effects of LPS-induced virulence factors on the brain and depression via the microbiota-gut-brain axis.

Two-component regulatory systems have been widely implicated in bacterial virulence. The two-component PhoP/PhoR system is a signal transduction pathway in both prokaryotes and eukaryotes. This system may serve as a general transduction system for the expression of genes involved in secondary metabolism. Many secondary metabolites in prokaryotes are repressed by a form of PhoP (i.e. a negative regulation) [39]. The PhoP/PhoR two-component system is also associated with the synthesis of virulence lipids and plays an important role in maintaining persistent infection in animal models [40,41]. Therefore, PhoP/R is considered as one of the key virulence factors [42]. BvrR/BvrS is the best characterized two-component regulatory system of *Brucella* and is essential for *Brucella abortus* virulence and regulates the expression of outer membrane proteins [43,44]. Our present study found that the bacteria containing genes for the virulence factors associated with the two-component regulatory systems, PhoP/PhoR and BvrR/BvrS, were greatly enriched in the gut microbiota of mice in the LPS group, suggesting that LPS treatment may promote secondary metabolite production, induce increased expression of virulence factors and enhance persistent infection ability of the gut microbiota. However, LETX-VI pretreatment may attenuate or even reverse the changes caused by LPS (Fig. 7b).

LPS, a virulence factor widely interacting with hosts, exerts a crucial role in regulating the interaction of bacteria with their host, can be recognized by host pattern-recognition receptors to initiate inflammation and immunity response and is the main cause of tissue degeneration and chronic damage [45,46]. The mutations of LPS can result in attenuated virulence [47,48]. When we used LPS to induce depressive behaviour by intraperitoneal injection, the abundance of the bacteria containing genes for virulence factor LPS increased dramatically, suggesting that LPS treatment may lead to an increase in the level of LPS in the gut. LETX-VI reduced the increased amplitude of the bacterial abundance. Furthermore, the flagellum has a role as a virulence factor and is needed for host-tissue penetration [49]. Drug resistance caused by the formation of biofilms is associated with the flagellum [50]. In our present study, although the effect of LPS on the bacteria with polar flagellar genes and thus maybe the level of polar flagella was not very obvious, LETX-VI still exhibited the inhibitory action on such LPS-caused changes (Fig. 7b).

### Correlation of differential virulence factors and metabolic pathway changes with depression phenotypes

**Fig. 8.**
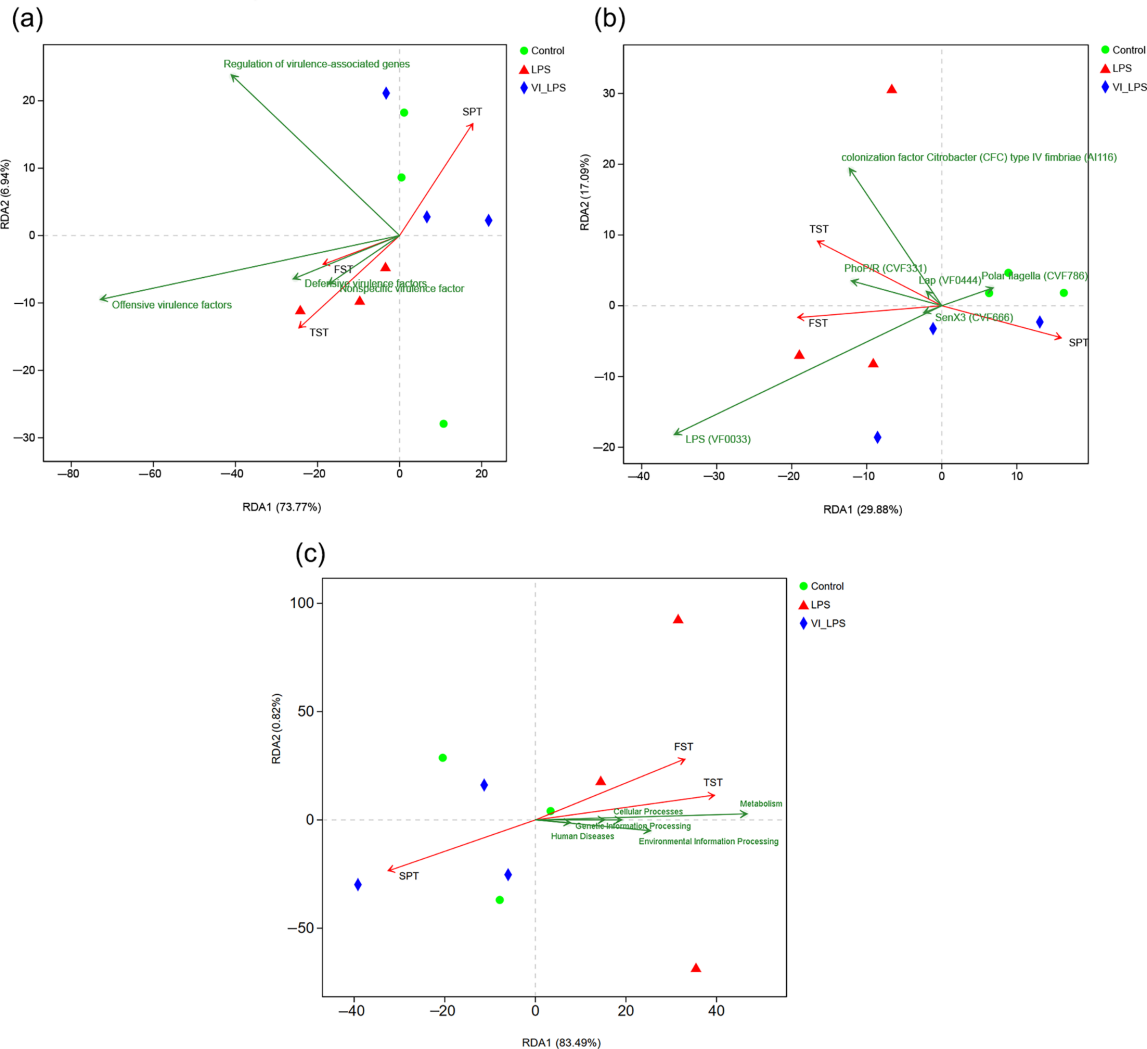
Correlation analysis between the differential virulence factors and metabolism pathway disturbance of mouse gut microbiota and the depression phenotypes. (**a**) RDA of the effects of different groups of virulence factors on depression phenotypes. (**b**) RDA of the correlation of differential virulence factors with depression phenotypes. (**c**) RDA of the correlation of metabolism pathway disturbance with depression phenotypes. LPS, LPS group; VI_LPS, LETX-VI-LPS group.

In order to further explore whether the differential virulence factors are correlated with the depression phenotypes of mice, redundancy analysis (RDA) was performed in this study. The analysis results showed that several groups of virulence factors, particularly offensive virulence factors, were closely positively correlated with the depression phenotype of FST and TST in mice and negatively correlated with that of SPT, whereas LETX-VI weakened the correlation of virulence factors with the depression phenotypes of mice (Fig. 8a). Among the differential virulence factors, the virulence factor LPS (VF0033), which prevents host surfactant proteins from clearing micro-organisms and protects bacteria from complement-mediated cell lysis [51,55], was most closely associated with the depression phenotypes of FST and TST (Fig. 8b). The correlation analysis between KEGG metabolic pathway and depression phenotypes showed that the metabolism pathway and human disease pathway were positively correlated with the depression phenotypes of mice FST and TST and negatively correlated with the depression phenotype of mice SPT. Moreover, compared with the human disease pathway, the metabolism pathway was more correlated with the depression phenotypes (Fig. 8c). These results suggest that the virulence factor accumulation and metabolic pathway disorder are significantly correlated with the LPS-induced depression phenotypes of mice; LETX-VI may have a certain preventive or reverse effect on the LPS-caused abnormal changes and thus improve the depression phenotypes.

### Correlation analysis between differential microbiota and depressive phenotypes

**Fig. 9.**
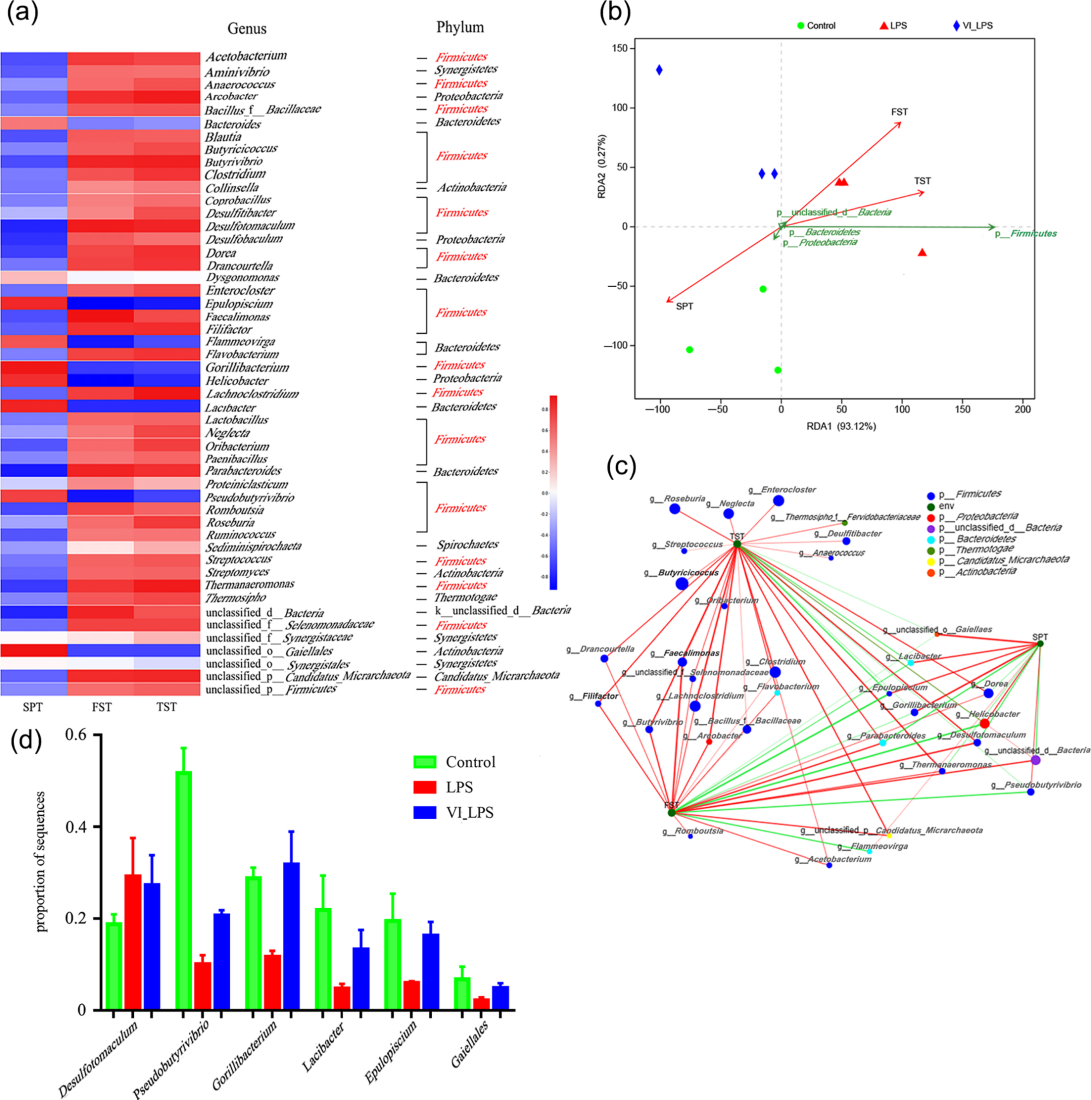
Correlation analysis between differential species of gut microbiota and depression phenotypes in mice. (**a**) Heat map analysis of the association between top 50 differential genera and depression phenotypes. (**b**) RDA of differential phyla and depression phenotypes. (**c**) The two-factor correlation analysis of differential genera and depression phenotypes. (**d**) LETX-VI pretreatment weakened the adverse effects of LPS on some depression-related bacterium species. LPS, LPS group; VI_LPS, LETX-VI-LPS group.

The heat map analysis was conducted on the correlation between the differential gut microbiota and depression phenotypes of the mice, and the results are shown in Fig. 9(a). Fig. 9(a) lists the top 50 genera whose abundance levels were mostly altered after LPS and LETX-VI-LPS treatments. It could be seen that, of the 50 genera, 31 belonged to *Firmicutes*, accounting for 62% of the top 50 differential genera, followed by *Bacteroidetes* containing 6 genera, accounting for 12% of the 50 genera. These data suggest that the disturbance of *Firmicutes* may be the main factor that led to the depression behaviours of the mice. In addition, the abundance alternations of the microbiotas shown in Fig. 9(a) demonstrated that LETX-VI could alleviate the LPS-induced changes in the abundance of some genera and phyla to a certain degree, which at least partially explains the mechanisms of LETX-VI improving the depression behaviours of the mice. Analysis of the correlation between the differential phyla and the depression phenotypes of mice showed that *Firmicutes* had a closely positive correlation with the depression phenotypes of the TST and FST and a negative correlation with SPT (Fig. 9b). A two-factor correlation network was constructed to further probe into the correlation between the differential genera and the depression behaviours of mice. The correlation network diagram visually showed that the genera belonging to *Firmicutes* were highly correlated with the depression phenotypes TST and FST (Fig. 9c). Through the correlation network diagram, some bacterial species highly associated with the three depression phenotypes were screened out, and the multi-group comparison was carried out. The results showed that, compared with the control, LPS induced an increase or decrease in the proportion of their sequences, and LETX-VI pretreatment could weaken the adverse effects of LPS on these depression-related bacteria (Fig. 9d). These results indicate that the gut microbiota in LPS-induced depression model mice is disrupted, and the disruption of the phylum *Firmicutes* is highly correlated with the depression phenotypes, and LETX-VI has a certain preventive and reverse effect on the disruption of the gut microbiota related to the depression phenotypes caused by LPS.

## Discussion

Accumulated research results have proved that gut microbiota can not only protect mucosa and participate in material transformation but also affect mood and regulate brain and behaviours via the microbiota-gut-brain axis. The imbalance of gut microbiota composition and function is one of the important reasons for the occurrence and progression of a variety of neurological diseases, including anxiety disorder and depression [56,60].

With the rapid development of next-generation sequencing and metagenomic technology, researchers have even more efficient ways to study the structural composition of gut microbiota [61,62]. In our present study, metagenomic sequencing of the gut microbiota of mice in the control, LPS and LETX-VI-LPS groups was performed, and the differences in the composition and function of gut microbiota were comprehensively analysed. Compared with the control, LPS-induced depression model mice showed obvious dysbiosis of gut microbiota, and the ratio of *Firmicutes* to *Bacteroidetes* (F/B) of gut microbiota significantly increased after LPS treatment. However, pretreatment of the mice with LETX-VI before LPS injection can significantly attenuate the dysbiosis of gut microbiota caused by LPS, reducing the F/B ratio significantly. A large number of studies have reported significant changes in the gut microbiota of patients with depression, but there are some inconsistencies in the analysis results due to different criteria for patient recruitment and data analysis methods; at the phylum level, the changes in the abundance of *Firmicutes* and *Bacteroidetes* are the focus of these studies [2,11, 63, 64].

The composition of microbiota in the gut of the host is closely related to the health of the host. For example, *Bacteroides* is the main butyric acid producer in the human gut [65]. Butyrate can inhibit the release of pro-inflammatory factors from macrophages and is an effective anti-inflammatory mediator [66,68]. *Bacteroides* also plays an important role in the production of *γ*-aminobutyric acid, an inhibitory neurotransmitter that can have a calming effect on neurons in the brain. Studies have shown that the abundance of *Bacteroides* is closely related to depression and inflammatory bowel disease [69,71]. *Pseudobutyrivibrio* is also a common gut beneficial bacterial genus that can produce beneficial substances such as butyric acid. Its increased abundance is beneficial to reduce gut inflammation and improve the symptoms of a variety of diseases [72]. In our present study, it was found that some butyric-producing bacterial genera, such as *Bacteroides* and *Pseudobutyrivibrio*, were abnormally reduced in the LPS group, and LETX-VI pretreatment had a significant inhibitory effect on the action of LPS. In addition, in the LPS-induced depression model mice, *Streptococcus* and other inflammatory bacteria were also significantly increased, among which *Streptococcus* belongs to pyococcus, which can cause various suppurative inflammation, meningitis and streptococcal allergic diseases, while the abundance of these species in the LETX-VI-LPS group was significantly reduced. These data suggest that the pathogenesis of depression is closely related to the regulation of the microbiota-gut-brain axis and the immune system.

The correlation analysis between the differential gut microbiota functions including virulence factors and the depression phenotypes of mice showed that the disorder of the metabolic pathway of microbiota and the change of virulence factors were highly correlated with the depression phenotypes of mice. The correlation analysis between the differential gut microbiota and the depressive phenotypes showed that the dysbiosis of *Firmicutes* induced by LPS was closely related to the depressive phenotypes, and LETX-VI pretreatment could attenuate these adverse effects of LPS on depression-related micro-organisms. These results indicate that the composition and function of gut microbiota in LPS-induced depression model mice are significantly disrupted and closely related to the depression phenotypes of mice, and the prevention and treatment effect of LETX-VI on depression behaviours of mice is significantly related to the regulation of gut microbiota composition and function.

## Conclusions

In the present study, LETX-VI pretreatment was shown to improve depression behaviour and prevent or attenuate the LPS-induced decrease in the ratio of *Firmicutes* to *Bacteroidetes* (F/B), number of pro-inflammatory bacteria (*P*<0.01) and butyric acid-producing bacteria in the gut microbiota (*P*<0.05). Furthermore, LETX-VI injection also alleviated the effect of LPS on the metabolic function of the gut microbiota and the abundance of bacteria encoding virulence factors (*P*<0.05). Association analysis showed that the changes in the composition and function of gut microbiota were closely related to the depression phenotypes of mice, suggesting that the abnormal function of gut microbiota is linked to depression. Taking all the observations in the present study into consideration, we propose that LETX-VI prevents depressive behaviour in an animal model by regulating the composition and function of the gut microbiota, which provides clues for the application of LETX-VI in the prevention and treatment of depression. However, the effects of LETX-VI on gut microbiota need further experimental confirmation, and the underlying mechanisms still need to be further investigated.

## Abbreviations

FST: forced swim test
LDA: linear discriminant analysis
LefSe: linear discriminant analysis effect size
LETX-VI: latroeggtoxin-VI
PCA: principal component analysis
RDA: redundancy analysis
SCH: schisandrin
SPT: sucrose preference test
TST: tail suspension test
